# Traumatic dislocation of the iris into the vitreous cavity with intact lens: a case report

**DOI:** 10.1186/s12886-023-03105-x

**Published:** 2023-08-21

**Authors:** Hai-Nan Xie, Lan-Lan Chen, Rui Wang, Zhi-Hong Zhu, Hou-Bin Huang

**Affiliations:** 1 Department of Ophthalmology, Hainan Hospital of Chinese PLA General Hospital, No 80. Jianglin Road, Haitang Bay, Sanya, 572013 China; 2 Senior Department of Ophthalmology, Chinese PLA General Hospital, Haidian District, No 28. Fuxing Road, Beijing, 100853 China; 3 The Second School of Clinical Medicine, Southern Medical University, NO 1023-1063 Shatai South Road, Guangzhou, 510280 Baiyun District China

**Keywords:** Traumatic aniridia, Penetrating ocular trauma, Iridodialysis, Artificial iris, Case report

## Abstract

**Background:**

Traumatic aniridia occurs when the iris is extruded from the eye and is often accompanied by lens injuries. However, traumatic aniridia due to dislocation of the iris into the vitreous cavity without lens damage has never been reported.

**Case presentation:**

A 30-year-old man presented with visual loss and pain for 6 h after a thin wire injured his right eyeball. Ophthalmologic examinations manifested a 2 mm full-thickness corneal laceration and total hyphema. An intact clear lens, healthy attached retina, and almost complete iris tissue in the vitreous cavity were found after resolution of hyphema the next day. Further examination revealed that the defect in the zonule below the corneal wound was the path for the iris to enter the vitreous cavity. The patient opted for nonsurgical treatment until pigment granules and opacity were observed in the vitreous cavity after 50 days. Vitrectomy was performed to remove the dislocated iris.

**Conclusions:**

The presentation of this unique case indicates that the torn iris was displaced to the vitreous cavity with an intact lens and missing local zonula instead of out the corneal laceration after a penetrating injury. The type of injury, mechanism, and force on the spot may contribute to the occurrence of this rare condition. Instead of artificial irises, tinted glasses were more appropriate treatment option for this patient. Peripheral retinal examination was essential in the management of this case. In such cases, the iris in the vitreous cavity should be resected to prevent complications.

## Background

Ocular trauma occurs in various situations and manifests in many ways with varying degrees of severity. The iris is a sensitive and delicate ocular structure that can be damaged by blunt, penetrating, or iatrogenic traumas. Traumatic iris injuries include iritis, mydriasis secondary to sphincter damage, iridodyalisis secondary to iris root injury, full or partial-thickness iris defects, and complete loss of the iris [1]. Traumatic iris injury results in glare, photophobia, reduced contrast sensitivity, and diminished esthetics depending on the extent and site of injury and severity of symptoms [2].

Penetrating injuries are primarily responsible for traumatic aniridia, which is often accompanied by lens and corneal lesions and can also occur in eyes with a history of cataract surgery or trabeculectomy [3]. However, there are no reports of traumatic iris displacement into the vitreous cavity without lens damage. Herein, we report a rare case with an exceptional presentation.

## Case presentation

**Fig. 1:**
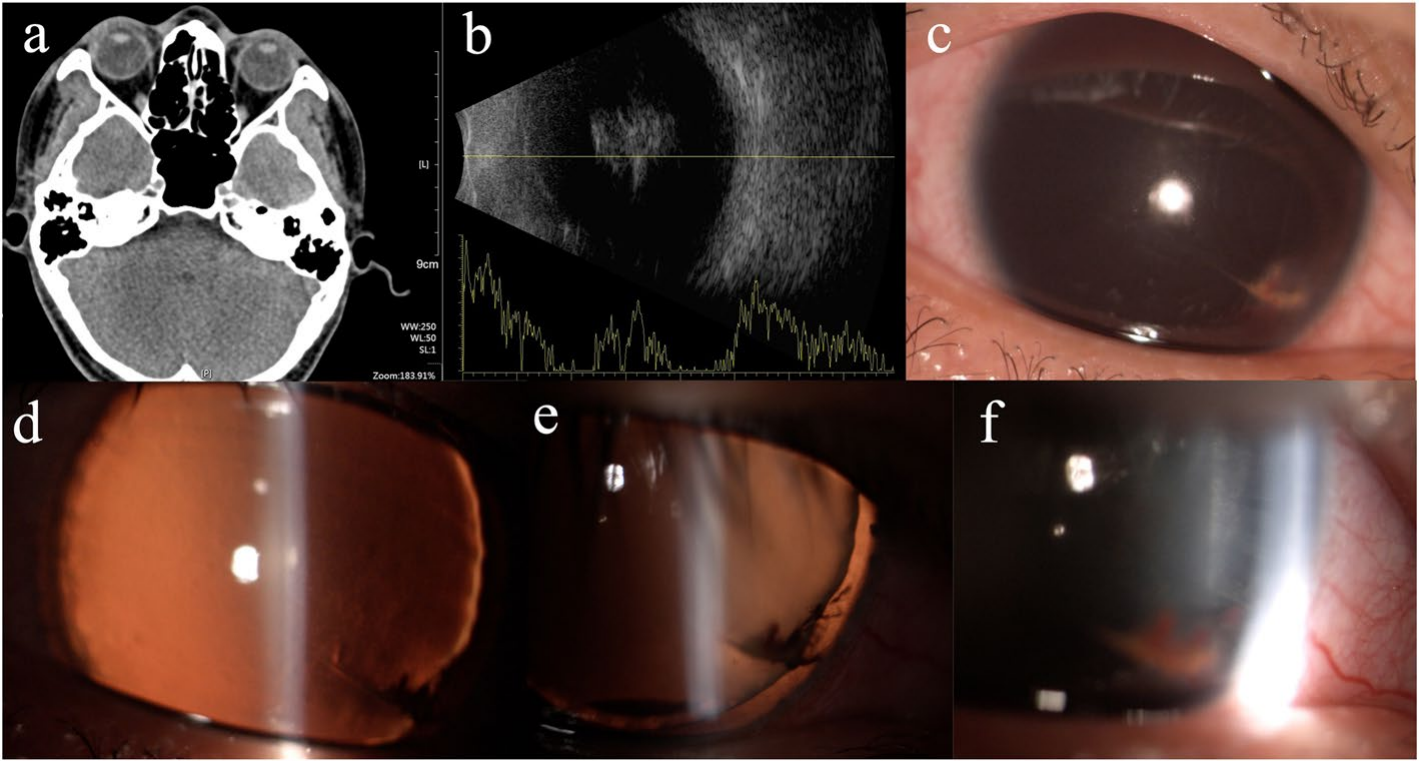
No intraocular foreign bodies were found on computed tomography scan **a** B-scan revealed mass opacity in the vitreous cavity 6 h after injured **b** 36 h after initial examination, a 2-mm full-thickness oblique laceration on the inferior-nasal aspect of the cornea **c** intact clear lens **d**, **e** zonules fibers **f** and complete loss of the iris are observed after resolution of the hyphema

A 30-year-old man presented with visual loss and pain for 6 h after a thin wire injured his right eyeball. The best-corrected visual acuity was hand movement at 10 cm, and intraocular pressure (IOP) was 26 mmHg in the right eye. Extraocular muscle movements and external examinations of both eyes were unremarkable. Slit-lamp microscopic examination revealed a 2-mm full-thickness oblique laceration on the inferior-nasal aspect of the cornea 2 mm from the limbus and complete hyphema. A Seidel test showed no leakage from the anterior chamber. B-scan revealed mass opacity in the vitreous cavity, possibly due to hemorrhage (Fig. 1). Computed tomography scan revealed no signs of injury and intraocular foreign bodies (Fig. 1). His past history and family history were unremarkable. Compound eye drops containing antibiotic and glucocorticosteroid were instilled and patch was applied to immobilize the traumatic eye.

**Fig. 2:**
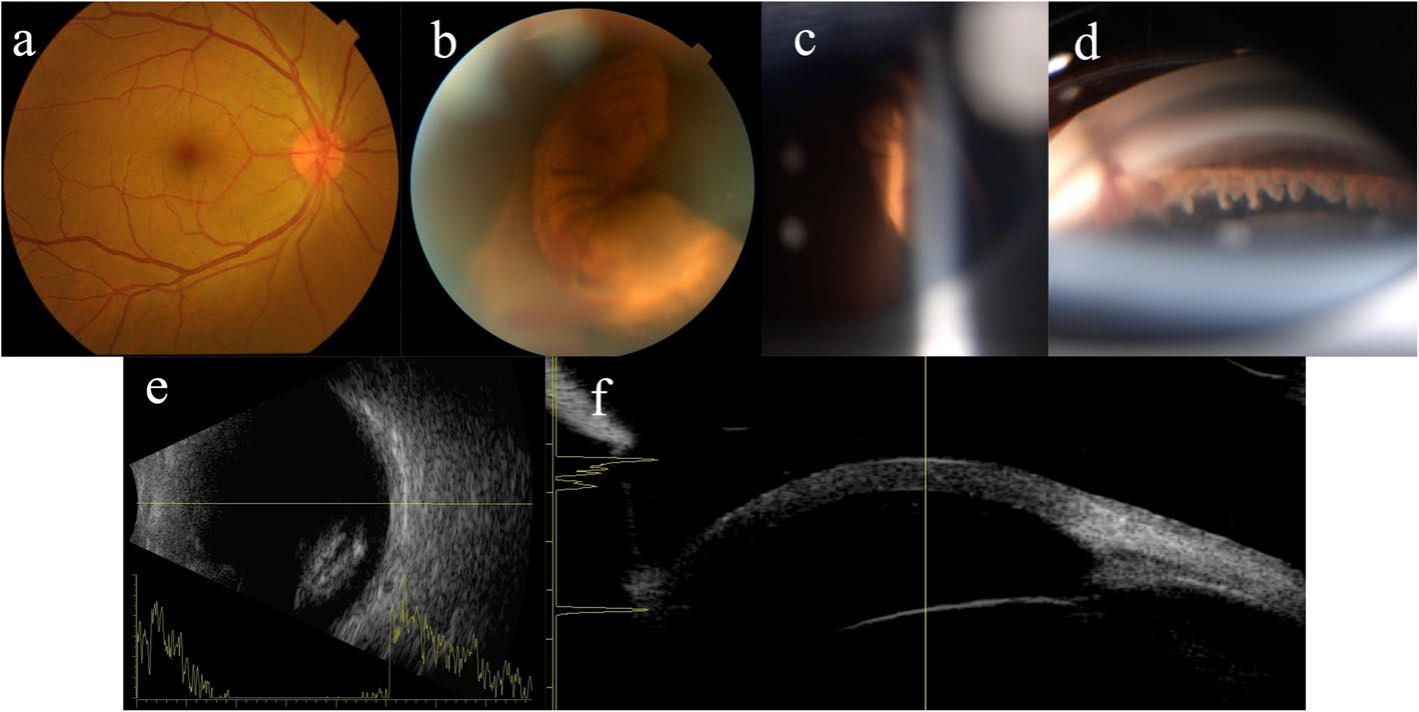
The posterior pole fundus was unremarkable **a** but brown iris tissue in the inferior-nasal field of the vitreous cavity was noted **b**, **c** which was detected by B-scan **e**. Gonioscopy revealed a clear view of the ciliary body and a hematocele band between the corneal endothelium and ciliary process below the laceration **d** Ultrasound biomicroscopy showed loss of iris, and no indication for cyclodialysis or angle recession **f**

A deep anterior chamber, intact clear lens, Zinn’s zonules, and complete loss of the iris were observed after to resolution of the hyphema 1 day after the initial examination (Fig. 1). Vision of the right eye improved to 0.3 Snellen and IOP improved to 14 mmHg. The dilated fundus examination showed trace hemocytes, healthy macula, and attached retina with intact vasculature. However, the presence of donut-like brown iris tissue in the inferior-nasal field of the vitreous cavity was noted (Fig. 2). Ultrasonography showed clumpy high echo for the dislocated iris, which was overlooked on the first scan for its insidious peripheral position (Fig. 2). Gonioscopy revealed a 360° absence of the iris, with a clear view of the ciliary body, and a hematocele band between the corneal endothelium and ciliary process below the laceration (Fig. 2). Ultrasound biomicroscopy showed no cyclodialysis or angle recession (Fig. 2). The patient's vision returned to 0.6 after 10 days of trauma.

Closer inspection with a slit lamp revealed a local loss of the zonule below the corneal laceration. This explained how the iris entered the vitreous cavity even when the transparent and intact lens was in situ. Medical history revealed that a steel wire with a diameter of less than 1 mm was bent when it was accidentally released, consequently propelling the wire into the eyeball and quickly bouncing off.

The patient’s vitreous chamber was unremarkable until 50 days later when opacities and pigment particles appeared in the vitreous cavity around the dislocated iris. The patient was treated with pars plana vitrectomy to remove the iris and prevent proliferative vitreoretinopathy and secondary glaucoma. Upon telephone follow-up 1 years after surgery, the patient has since maintained a visual acuity of 0.6 with mild photophobia. Artificial iris implantation was recommended. However, this recommendation was not complied with because of the operative risk that accompanies the procedure. Aniridia does not interfere with his daily life since the patient works indoors and goes out on sunny days with sunglasses.

## Discussion and conclusions

According to the Birmingham Eye Trauma Terminology System, mechanical eye trauma can be divided into two categories: closed and open globe injuries [4]. Iris injuries have various clinical presentations in ocular trauma, including iris sphincter ruptures, iritis, iridodialysis, iris defects, and aniridia. They are often associated with hyphema due to damage to the iris vessels. Hyphema may be short-lived and resolve spontaneously. However, it can be complicated by secondary glaucoma and corneal blood staining. Traumatic iritis and iris sphincter rupture frequently develop after a closed-globe injury. Although closed-globe injury is also primarily responsible for iridodialysis and iris defects, it may also be associated with penetrating injuries [5, 6].

Ocular penetrating injuries frequently result in exposure or extrusion of intraocular contents due to internal and external pressure differences in the eyeball. In some instances of corneal or corneoscleral laceration, the iris can undergo root avulsion from the ciliary body, leading to iridodialysis, aniridia, and dark brown iris tissue incarcerated in the wound. Expulsive iridodialysis or traumatic aniridia often accompany other injuries, such as lens dislocation, cataract, commotio retinae, and retinal detachment [3, 6]. The expulsed iris may or may not be identified during surgery, as blood in the anterior chamber often prevents adequate visualization of the anterior segment. During surgery, they are brought back into the eyeball provided that they are not dirty, desiccated, or necrotic.

Ocular lacerations may be caused by sharp objects, such as glass, wire, or the blade of a knife or scissors. Traumatic wound closure occurs automatically if the wound is small and without incarcerated intraocular contents. In our case, the trauma caused a single corneal wound without a retained intraocular foreign body, and the retina was not damaged. The corneal incision was closed, and the iris was torn from the ciliary body and displaced into the vitreous chamber while sparing the lens. The lens was intact and central in situ, and only partial zonular fiber loss was observed. Gonioscopy revealed a hematocele band between the corneal endothelium and the ciliary process below the laceration. We inferred that there are three critical points in this case. First, the steel wire entered the eyeball from the temporal side according to the contour of the corneal laceration, and the wire tip was blunt, which was confirmed by the patient; therefore, it did not penetrate the local iris. Second, the patient was young and had good iris elasticity. Third, the strength and position of the force point between the wire and the iris were unique. These factors determined that the steel wire could exactly press the iris to form a complete iris avulsion rather than iridodialysis or iris perforation. The steel wire carried the iris into the vitreous cavity through the gap between the lens and the ciliary process. It exited instantaneously, leaving the iris in the vitreous cavity.

Several cases of traumatic aniridia with simultaneous intraocular lens and capsular bag preservation in eyes with a history of cataract extraction have been reported [7–10]. The researchers, therefore, proposed that the impact caused the original corneal tunnel to open, and the iris tissue was completely dislodged and expelled through a clear corneal surgical incision. The tunnel was then closed, and the globe appeared intact upon examination. Owing to the characteristics of the pigmented membrane, the iris was ejected through the corneal incision resulting in a sudden increase in intraocular pressure due to blunt trauma that allowed it to pass through the gap between the lens and ciliary body caused by mechanical pressure.

There have been reports of prolapsed iris tissue superiorly through trabeculectomy scleral flap wounds, resulting in aniridia with an intact capsular bag and lens held by zonular fibers, and pigmentation that may become lighter in degree over time. Kaliperuma et al. reported a case of a patient who presented 4 months after trauma, wherein the subconjunctival pigmentation was mild and limited to the superior fornix [11, 12]. Yang et al. reported a case of traumatic aniridia with extensive pigmentation in the episclera after hitting his left eyeball on a table corner [3]. The patient had undergone trabeculectomy and phacoemulsification many years prior to the accident. They concluded that the scleral fistula served as a less resistant point for releasing pressure than a healed corneal wound when the eye encounters a contusion injury. Our case showed vitreous opacity and pigment particles around the dislocated iris 50 days after injury. It is likely that pigments stimulate proliferative vitreoretinopathy and angle occlusion in secondary glaucoma if untreated. Similarly, when displaced into the vitreous cavity, the iris is likened to a special intraocular foreign body that should be removed as soon as possible.

Treatment options of aniridia consisted of colored contact lenses, sunglasses, corneal stromal implants, corneal tattooing, and prosthetic iris devices [7, 13]. Many studies have reported that the use of artificial iris implants is an effective option for treating extensive traumatic iris defects for the reconstruction of the anterior segment, good functional outcomes, and high patient satisfaction [14]. Some implants were made of foldable silicone material, which reduces the need for large incisions, and custom‐made accounts for a wide range of applications. However, artificial iris prostheses still have limitations and complications, such as uveitis, glaucoma, band keratopathy, corneal endothelial damage, and iris implant dislocation [15]. Each complication must be addressed to prevent irreversible blindness. The development of high IOP is one of the main issues and is consistent with a range of 46.1%-71.4% reported in previous studies [16, 17]. The underlying mechanisms of glaucoma may include pressure of the edges of the prosthesis on the ciliary body, causing cyclitis, mechanical trabecular injury, trabeculitis, peripheral anterior synechiae, and angle-closure glaucoma [16].

The lens must be removed if the functional prosthetic iris device is implanted because it is not indicated for implantation into eyes with clear crystalline lenses. Our patient did not undergo artificial iris implantation because of current visual acuity and possible postoperative complications, and the aniridia did not cause much inconvenience to activities of daily living. As for the patient, tinted glasses were deemed to be safer and more reliable than artificial irises. However, simultaneous implantation of the intraocular lens and the artificial iris maybe a more appropriate option if vision-threated complicated cataract develop due to the vitrectomy surgery.

## Data Availability

The datasets used and/or analyzed during the current study are available from the corresponding author upon reasonable request.

## Abbreviation

IOP: Intraocular pressure
